# Bis[1,2-bis­(3,5-di­methyl­phen­yl)ethyl­ene-1,2-di­thiol­ato(1−)]nickel(II)

**DOI:** 10.1107/S2056989025004293

**Published:** 2025-05-20

**Authors:** Titir Das Gupta, Jackson Guite, LiWen Hirt, Xiaodong Zhang, James P. Donahue

**Affiliations:** a Department of Chemistry, Tulane University, 6400 Freret Street, New Orleans, Louisiana 70118-5698, USA; Texas A & M University, USA

**Keywords:** crystal structure, di­thiol­ene, nickel, electron-donating, C—H→π_arene_ hydrogen bonds

## Abstract

The title compound, [Ni(C_18_H_18_S_2_)_2_], **1**, crystallizes upon an inversion center in monoclinic *P*2_1_/*c* with intra­ligand C—S and C—C_chelate_ bond lengths that are indicative of the radical monoanionic redox level for the di­thiol­ene ligand. The arene rings are canted at angles of 43.54 (4) and 71.36 (3)° with respect to the NiS_4_ plane. Mol­ecules of **1** form columnar stacks along the *a* axis of the cell with a ∼59° angle between the mol­ecular plane and the stacking axis. Adjacent stacks alternate in the directionality of their cant relative to the stacking axis such that a 37.2° angle is defined by the core NiS_4_ planes of mol­ecules from adjoining columns. Inter­molecular inter­actions are governed by methyl C—H→π_arene_ hydrogen bonds, with each mol­ecule enjoying four such inter­actions as acceptor and providing four such inter­actions as donor.

## Abstract 2

Square-planar bis­[1,2-bis­(3,5-di­methyl­phen­yl)ethyl­ene-1,2-di­thiol­ato(1–)]nickel(II) crystallizes on an inversion center in monoclinic *P*2_1_/*c* in a packing arrangement defined by a dense network of inter­molecular methyl C—H→π_arene_ hydrogen bonds.

## 1. Chemical context

First synthesized in the middle 1960s, transition-metal di­thiol­ene complexes elicited inter­est initially because their electronic structures did not conform to classical descriptive formalisms and led to the recognition that complexes with redox-active ligands define a category of coordination compounds that distinctively differ from those with metal-based frontier MOs (Eisenberg & Gray, 2011 ▸). For di­thiol­ene complexes of the Group 10 metals particularly, their potential as novel, engineered materials for a broad range of applications has motivated steady effort aimed at the development of new synthetic methods and at characterization of their optical, electronic, and magnetic properties. Examples of the important behavior manifested by this compound class include the reversible bleaching of nickel di­thiol­ene dyes under intense radiation (Mueller-Westerhoff *et al.*, 1991 ▸), superconductivity in mol­ecular Group 10 bis­(di­thiol­ene) complexes (Kato, 2004 ▸), sensing of organophosphate toxins by platinum di­thiol­ene 1,2-bis­(di­phenyl­phosphino)ethane complexes (Van Houten *et al.*, 1998 ▸), ferromagnetism (Robertson & Cronin, 2002 ▸; Faulmann & Cassoux, 2004 ▸), and catalytic H_2_-evolution by nickel bis­(di­thiol­ene) complexes under photolysis (Zarkadoulas *et al.*, 2016 ▸). Because the redox, electronic, and optical properties that support many of these applications are directly and rationally influenced by the chemical nature of the di­thiol­ene substituents, the development and characterization of new ligand derivatives and their complexes with Group 10 metals is of continuing inter­est. In this report, we detail the synthesis and crystal structure of nickel bis­(3,5-di­methyl­phen­yl)di­thiol­ene, a coordination compound and a di­thiol­ene ligand variant that have not previously been reported.
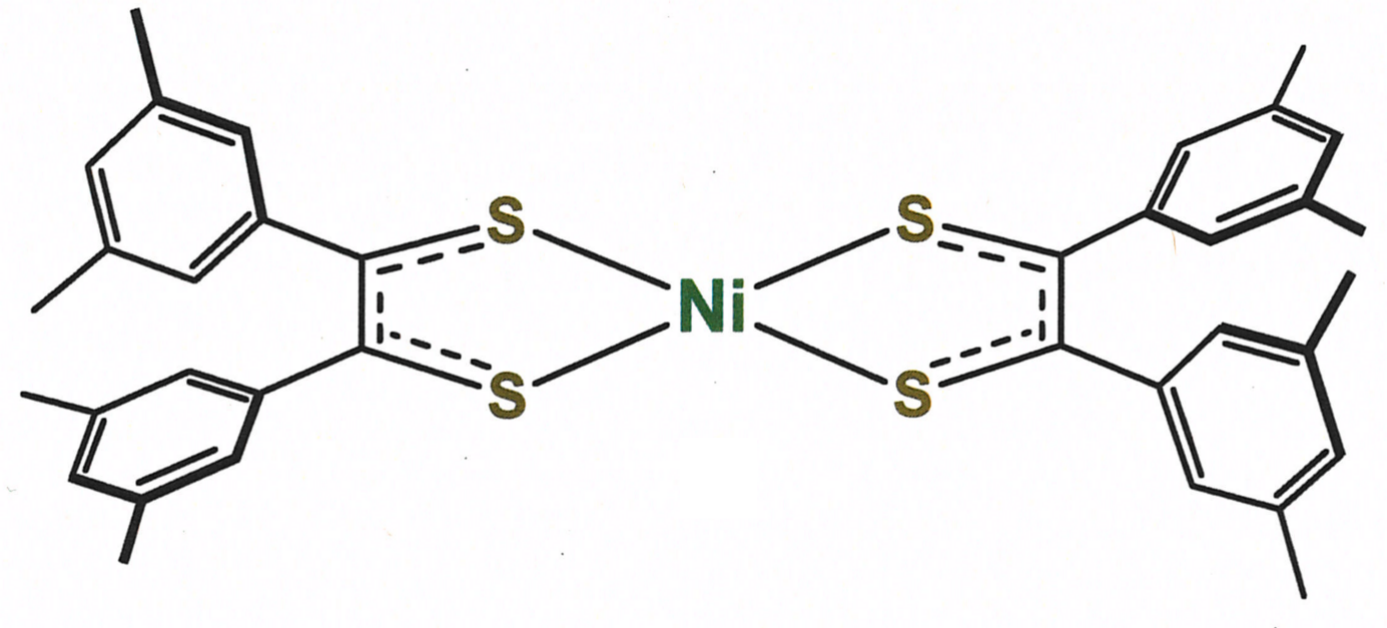


## 2. Structural commentary

Bis[1,2-bis­(3,5-di­methyl­phen­yl)ethyl­ene-1,2-di­thiol­ato(1−)]nickel(II), **1**, is prepared in 38% yield using the phospho­rus sulfide-benzil/benzoin protocol first disclosed by Schrauzer (Schrauzer & Mayweg, 1965 ▸). The separation of **1** from the solution as a microcrystalline precipitate is enabled by its hydro­carbon-rich periphery, its centrosymmetry and the polar, mixed aqueous-organic medium in which it forms. Its purity at this point is sufficient for most further purposes, but it is readily tractable to preparative-scale recrystallization by vapor diffusion methods.

**Figure 1:**
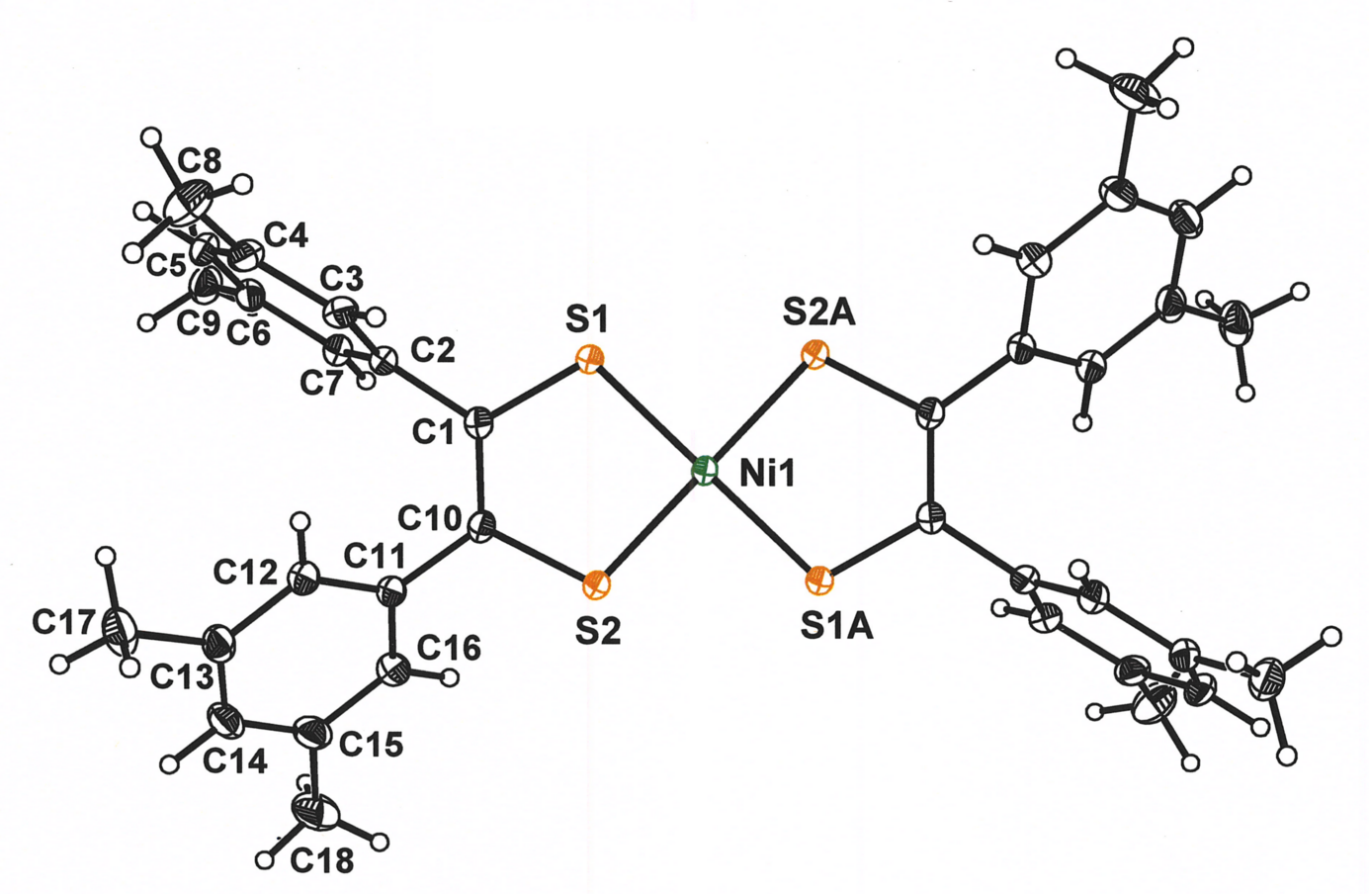
Displacement ellipsoid plot of **1** at the 50% probability level with atom labeling. Symmetry-related atoms other than S1*A* and S2*A* are not labeled. Symmetry code: (*A*) −*x*, 2 − *y*, 1 − *z*.

**Figure 2:**
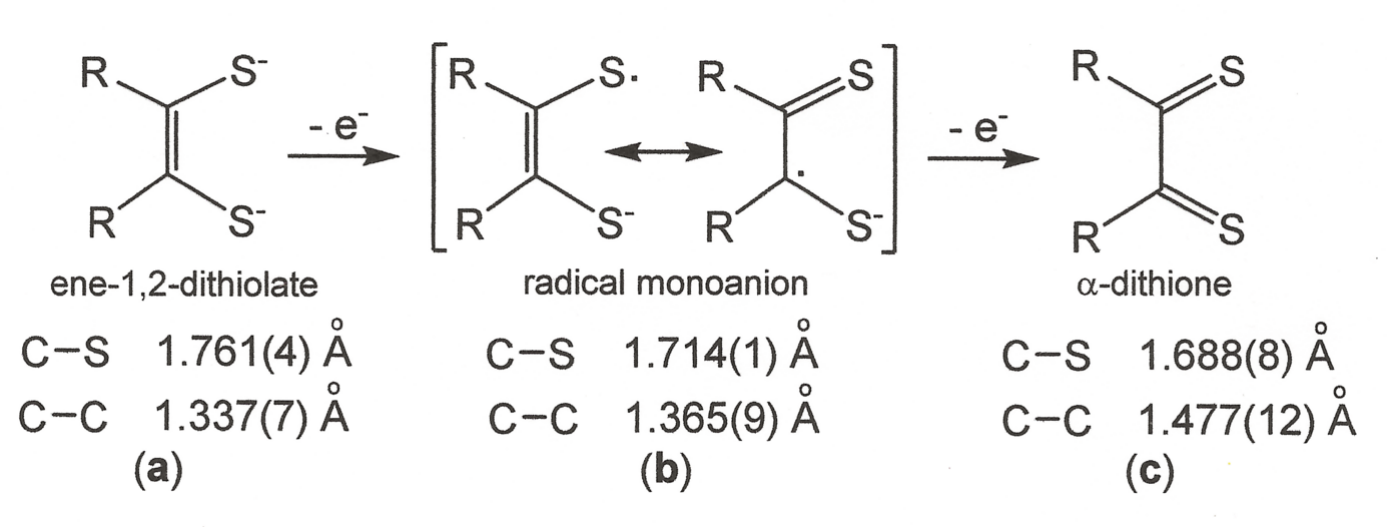
Redox levels of the di­thiol­ene ligand shown with the S—C and C—C intra-ligand bond lengths that are typical for each redox state. The S—C and C—C bond lengths decrease and increase, respectively, as the redox series is traversed from ene-1,2-di­thiol­ate(2–) (**a**) to α-di­thione (**c**).

The NiS_4_ inter­ior of **1** is rigorously planar, a feature necessitated by the occurrence of the mol­ecule upon a crystallographic inversion center (Fig. 1 ▸). The placement of the Ni^2+^ ion upon an inversion center has the additional consequence that only half of the coordination complex is unique. The angles at which the arene substituents join the C_2_S_2_Ni chelate ring are 43.54 (4) and 71.36 (3)°. The 1.7063 (8) Å S—C_ave_ and 1.393 (2) C—C_chelate_ bond lengths in **1** are similar to the 1.714 (1) and 1.365 (9) Å observed for the corresponding bonds in Me_2_C_2_S**^.^**S^−^ in [Ni(S_2_C_2_Me_2_)_2_] (Fig. 2 ▸; Lim *et al.*, 2001 ▸). This fact, in conjunction with the observation that these inter­atomic distances lie between those for the C—S and C—C_chelate_ bonds in [Ni(S_2_C_2_Me_2_)_2_]^2–^ (Lim *et al.*, 2001 ▸) and [Ni((S=C)_2_(NMeCH_2_)_2_)_2_]^2+^ (Bigoli, *et al.*, 2001 ▸), in which the ligand redox levels are fully reduced ene-1,2-di­thiol­ate and fully oxidized α-di­thione, respectively, affirms a radical monoanionic description of the ligands in **1** (*cf.* Fig. 2 ▸).

## 3. Supra­molecular features

**Figure 3:**
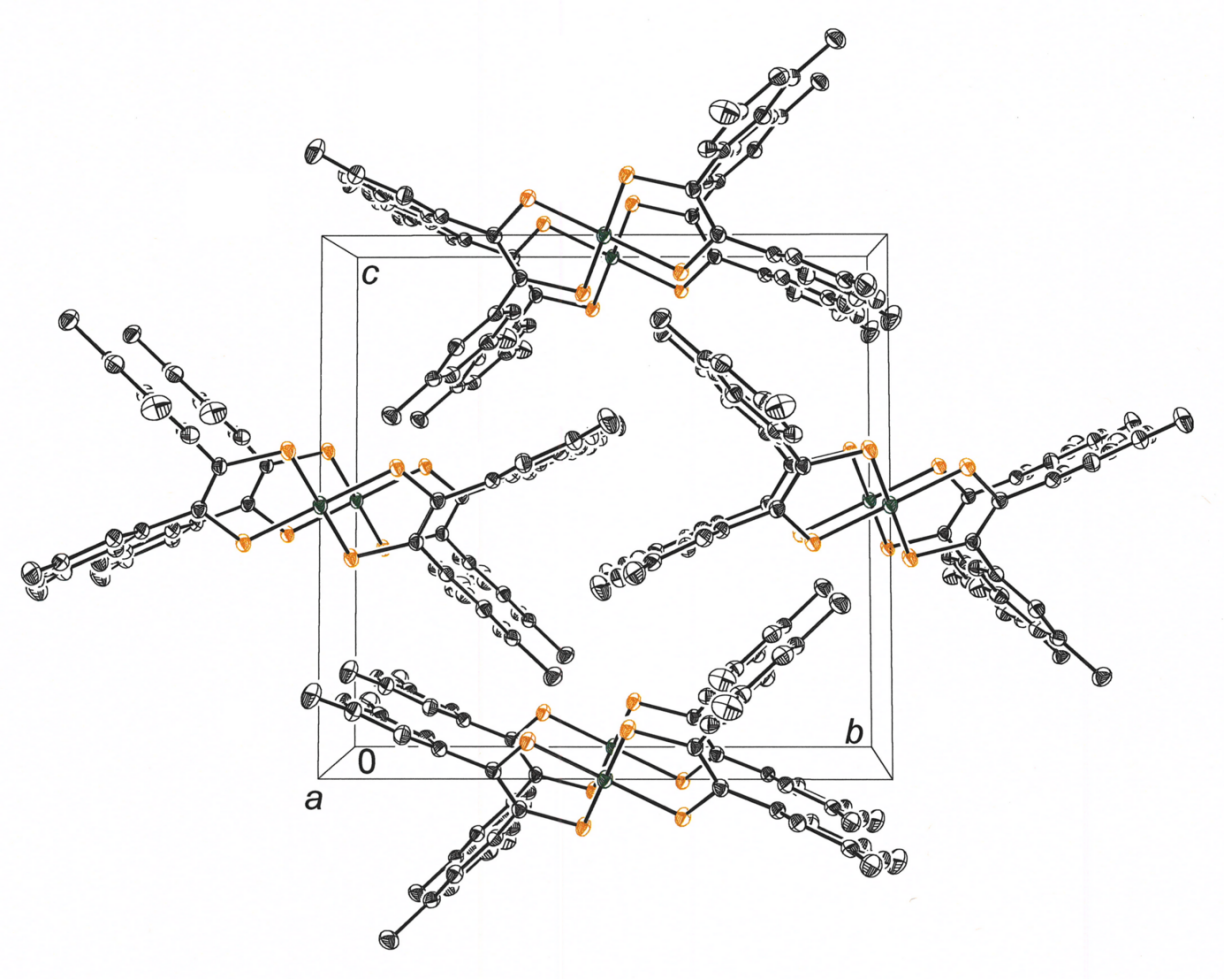
View along the *a* axis of the unit cell, revealing columnar stacks of **1**. Displacement ellipsoids are shown at the 50% level, and all H atoms are omitted for clarity.

**Figure 4:**
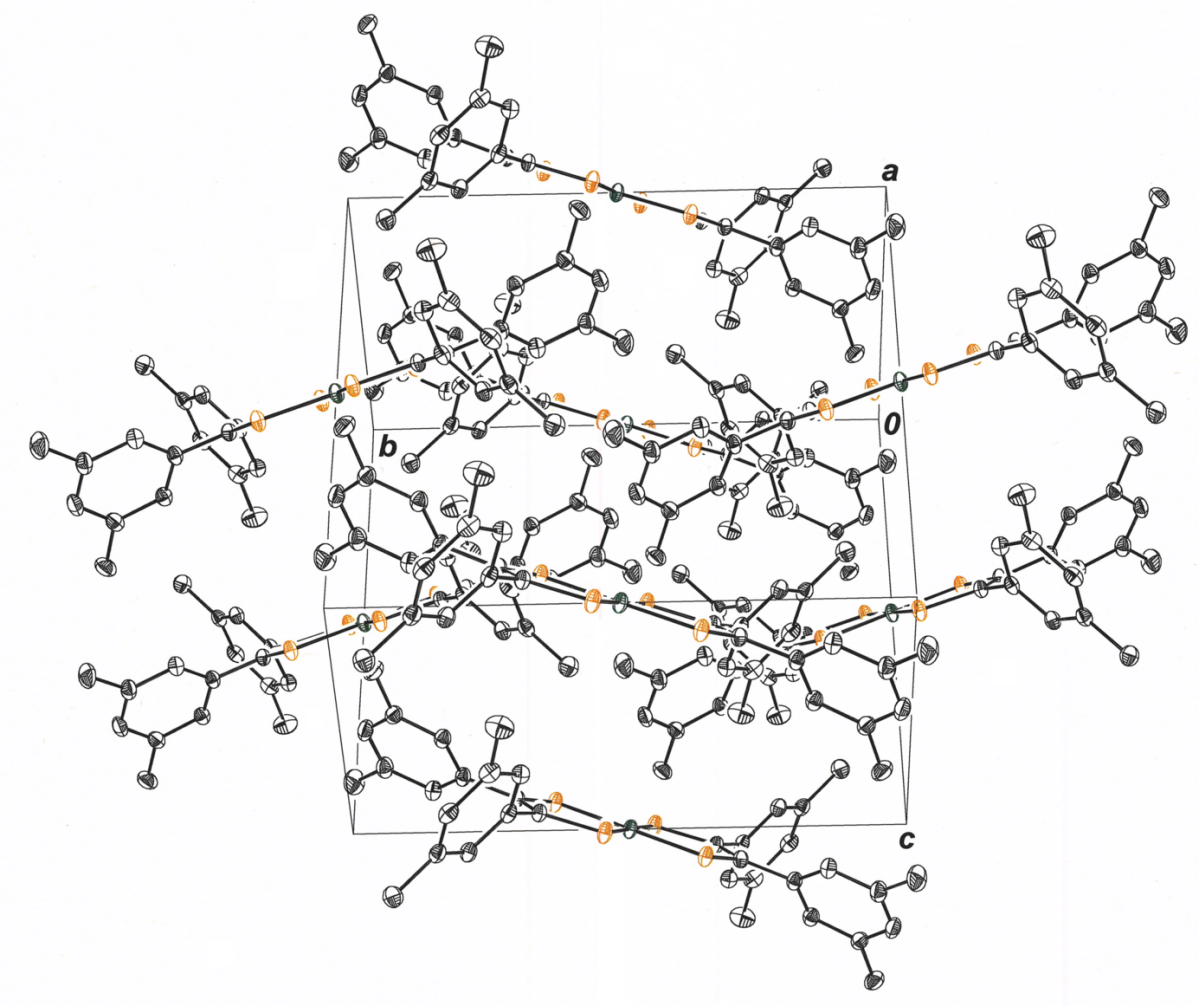
A ‘side-on’ perspective of the columnar stacks of **1** illustrating the alternating orientation of the mol­ecular plane relative to the stacking axis. The view is approximately along the direction of the *ac* face diagonal of the cell. Displacement ellipsoids are drawn at the 50% level, and all H atoms are omitted for clarity.

**Figure 5:**
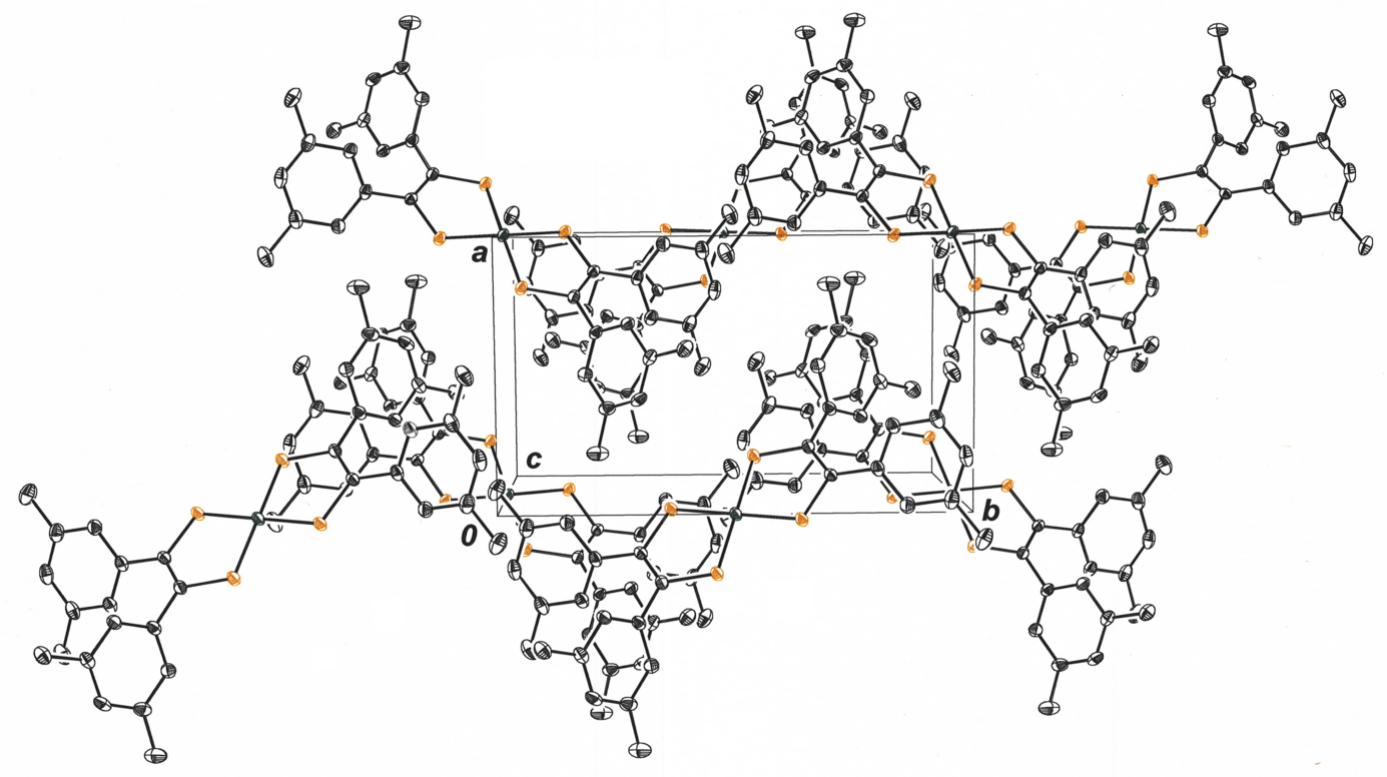
View of the mol­ecular packing for **1** along the *c* axis of the unit cell. A sawtoothed appearance to the packing motif is again evident but with more acute angle in the pattern. Displacement ellipsoids are drawn at the 50% level, and all H atoms are omitted for clarity.

The packing arrangement places mol­ecules of **1** into columnar stacks along the *a-*axis direction of the cell with aryl rings of neighboring mol­ecules forming both parallel planar and near orthogonal inter­actions (Fig. 3 ▸). When adjacent columns of mol­ecules are viewed from the perspective of the edge of the NiS_4_ plane (Fig. 4 ▸), an alternation is evident in the disposition angle of the mol­ecular plane with respect to the stacking axis. The centermost stack in Fig. 4 ▸ reveals mol­ecules whose planar core is rotated clockwise approximately 31° from orthogonality to the stacking axis, while the adjoining columns of **1** have the mol­ecular plane inclined in the opposite direction by an equal magnitude. Regardless of the directionality of the tilt, the mol­ecular planes form an angle of about 59° with the *a* axis. From the vantage point presented in Fig. 4 ▸, mol­ecules from neighboring stacks are canted at a 37.2° angle relative to one another such that a herringbone-like motif to the packing arrangement is created. A perspective view down the *c* axis of the cell again shows a zigzag pattern formed as rows of **1** related by simple translation along *b* project onto other rows of **1** that are canted in the other direction as they are also replicated by translation along *b* (Fig. 5 ▸).

**Figure 6:**
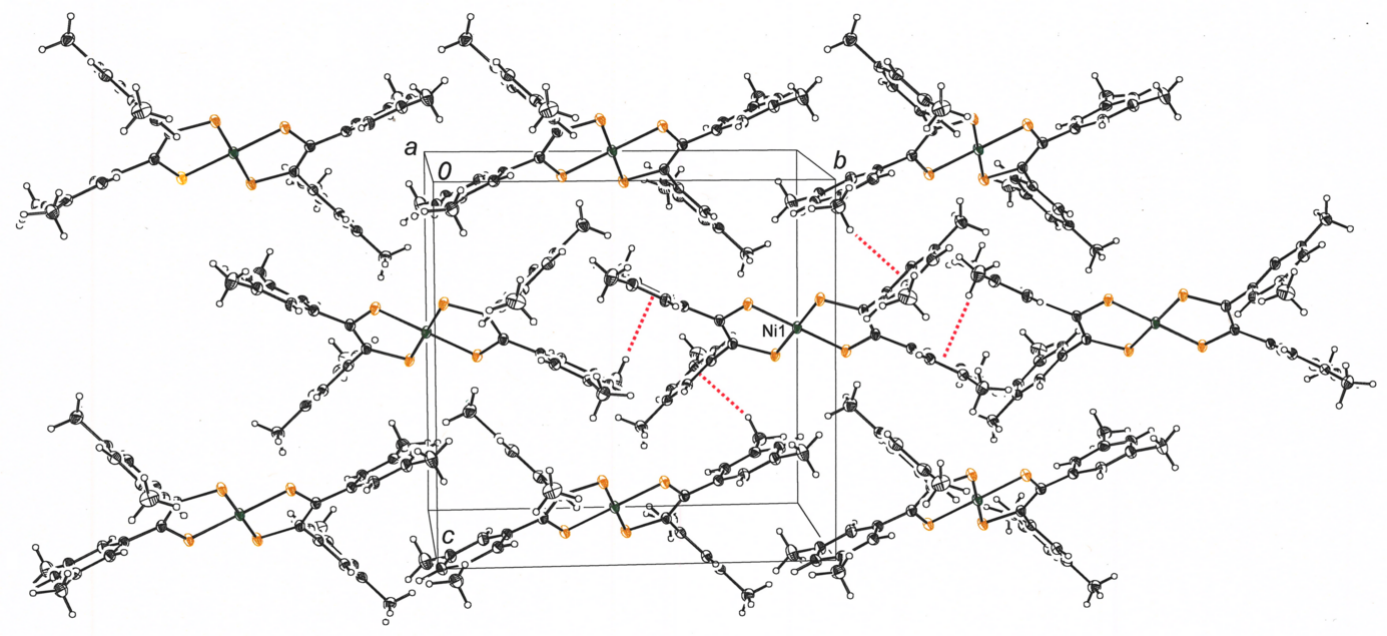
Packing arrangement of **1** in rows along the *b* axis (left-to-right) as viewed down the *a* axis of the cell. The four C–H → π_arene_ inter­actions in which the mol­ecule with the Ni1 label serves as acceptor are illustrated with heavy dashed red lines. Displacement ellipsoids are presented at the 50% level.

Inter­molecular inter­actions between stacks are governed by methyl C—H→π_arene_ non-classical hydrogen bonds rather than by aliphatic dispersion forces. The distance between the C2–C7 ring centroid and H17*C* of an adjoining mol­ecule is 2.97 Å, while H18*B* from a different neighboring mol­ecule is positioned 2.87 Å from the C11–C16 ring centroid. This pattern is replicated on the mol­ecule’s other side such that each mol­ecule functions as an acceptor of four such C—H→π_arene_ hydrogen bonds, one with each 3,5-di­methyl­phenyl ring (Fig. 6 ▸). The heavy red dashed lines that depict these inter­actions evoke a windmill-like symmetry in Fig. 6 ▸. Additionally, both H17*C* and H18*B* from each end of **1** serve as donors of C—H→π_arene_ hydrogen bonds to adjacent mol­ecules, providing each mol­ecule of **1** with an additional four C—H→π_arene_ hydrogen bonds as C—H donor. While such inter­actions are individually weak with an inter­action strength in the range of 1.5–2.5 kcal mol^−1^ (Nishio, 2012 ▸), their collective effect appears to be decisive in guiding the inter­leaved arrangement of 3,5-di­methyl­phenyl rings.

## 4. Database survey

The bis­(3,5-di­methyl­phen­yl)di­thiol­ene ligand does not appear in any structurally characterized coordination compound of the transition metals or any other element (CSD, Version 2024.3; Groom *et al.*, 2016 ▸), nor does any report of its use occur in other information databases. The set of structurally characterized homoleptic nickel bis­(di­thiol­ene) complexes with aryl-type substituents, [Ni(S_2_C_2_Ar_2_)_2_], to which **1** is now joined includes those where Ar = Ph (Megnamisi-Belombe & Nuber, 1989 ▸; Kuramoto & Asao, 1990 ▸; Miao *et al.*, 2011 ▸), Me-4-C_6_H_4_ (Miao *et al.*, 2011 ▸), ^*t*^Bu-4-C_6_H_4_ (Das Gupta *et al.*, 2023 ▸), Cl-4-C_6_H_4_ (Koehne *et al.*, 2022 ▸), MeO-4-C_6_H_4_ (Arumugam *et al.*, 2007 ▸), and 3,5-(MeO)_2_-4-^*n*^BuO-C_6_H_2_ (Nakazumi *et al.*, 1992 ▸). Among these complexes, the largest twist angle between an aryl ring substituent and the NiS_4_C_4_ plane is 65.77° in the monoclinic polymorph of [Ni(S_2_C_2_Ph_2_)_2_] (Miao *et al.*, 2011 ▸), a value that is appreciably less than the 71.36 (3)° twist angle in **1**. While C—H→π_arene_ hydrogen-bond inter­actions are very apparent in this *P*2_1_/*n* form of [Ni(S_2_C_2_Ph_2_)_2_], close approach of H atoms to the ring centroids of neighboring mol­ecules easily occurs with lessened rotation of the aryl substituent from the planar core.

## 5. Synthesis and crystallization

Phospho­rus sulfide (P_4_S_10_, 0.379 g, 0.853 mmol) and 1,2-bis­(3,5-di­methyl­phen­yl)ethane-1,2-dione (0.353 g, 1.32 mmol) were dissolved in 1,4-dioxane (50 ml) and refluxed under a N_2_ atmosphere for 3.5 h. The resulting mixture was cooled to ambient temperature, filtered to remove unreacted solids, transferred to a solution of [Ni(OH_2_)_6_]Cl_2_ (0.129 g, 0.541 mmol) in deionized H_2_O (5 ml) and brought to reflux again for 3 h under N_2_. Upon cooling, the reaction mixture deposited crude **1** as a dark microcrystalline solid, which was collected by vacuum filtration, washed in succession with portions of deionized H_2_O, EtOH, and Et_2_O and then dried overnight under vacuum. Yield: 0.135 g, 0.206 mmol, 38%. Diffraction quality, prism-shaped black crystals were grown by the diffusion of MeOH vapor into a benzene solution. ^1^H NMR (δ, p.p.m. in CDCl_3_): 7.00 (*s*, 3 H, *ortho* and *para* aromatic C—H), 2.25 (*s*, 24 H, –CH_3_). ^13^C NMR (δ, p.p.m. in CDCl_3_): 181.9, 141.3, 137.8, 130.6, 126.9, 21.3. UV-vis [CH_2_Cl_2_, λ_max_, nm (ɛ_M_, *M*^−1^·cm^−1^)]: 270 (11800), 320 (15000), 605 (670), 875 (10200). Cyclic voltammetry (CH_2_Cl_2_, [^*n*^Bu_4_N][PF_6_] supporting electrolyte, Cp_2_Fe^+^/Cp_2_Fe as reference): **1** – e^−^ → [**1**]^+^, +0.611 V; **1** + e^−^ → [**1**]^−^, −0.509 V; [**1**]^−^ – e^−^ → [**1**]^2–^, −1.323 V. Analysis calculated for C_36_H_36_S_4_Ni: C, 65.95; H, 5.53; S, 19.56. Found: C, 65.86; H, 5.49; S, 19.49.

## 6. Refinement

Crystal data, data collection and structure refinement details are summarized in Table 1 ▸. Hydrogen atoms were added in calculated positions and refined with isotropic displacement parameters that were approximately 1.2 times (for aromatic C—H) or 1.5 times (for –CH_3_) those of the carbon atoms to which they were attached. The C—H distances assumed were 0.95 and 0.98 Å for the aromatic C—H and –CH_3_ types of hydrogen atoms, respectively. Rotation around the CH_3_—C_aromatic_ bonds was used to identify the positional variant that best modeled the experimental electron-density map.

## Supplementary Material

Crystal structure: contains datablock(s) I, global. DOI: 10.1107/S2056989025004293/jy2060sup1.cif

Structure factors: contains datablock(s) I. DOI: 10.1107/S2056989025004293/jy2060Isup2.hkl

CCDC reference: 2450503

Additional supporting information:  crystallographic information; 3D view; checkCIF report

## Figures and Tables

**Table 1:** Experimental details

Crystal data	
Chemical formula	[Ni(C_18_H_18_S_2_)_2_]
*M*_r_	655.60
Crystal system, space group	Monoclinic, *P*2_1_/*c*
Temperature (K)	150
*a*, *b*, *c* (Å)	8.4446 (2), 14.0911 (2), 13.5880 (2)
β (°)	96.133 (1)
*V* (Å^3^)	1607.63 (5)
*Z*	2
Radiation type	Cu *K*α
μ (mm^−1^)	3.47
Crystal size (mm)	0.14 × 0.14 × 0.04

Data collection	
Diffractometer	Bruker D8 QUEST PHOTON 3 diffractometer
Absorption correction	Multi-scan (*SADABS*; Krause *et al.*, 2015 ▸)
*T*_min_, *T*_max_	0.77, 0.88
No. of measured, independent and observed [*I* > 2σ(*I*)] reflections	33748, 3139, 2827
*R*_int_	0.077
(sin θ/λ)_max_ (Å^−1^)	0.618

Refinement	
*R*[*F*^2^ > 2σ(*F*^2^)], *wR*(*F*^2^), *S*	0.024, 0.066, 1.08
No. of reflections	3139
No. of parameters	191
H-atom treatment	H-atom parameters constrained
Δρ_max_, Δρ_min_ (e Å^−3^)	0.29, −0.30

Computer programs: *APEX4* and *SAINT* (Bruker, 2021 ▸), *SHELXT* (Sheldrick, 2015*a* ▸), *SHELXL2018/1* (Sheldrick, 2015*b* ▸) and *SHELXTL* (Sheldrick, 2008 ▸).

## supplementary crystallographic information

### Bis[1,2-bis(3,5-dimethylphenyl)ethylene-1,2-dithiolato(1-)]nickel(II) Crystal data

**Table:**

[Ni(C_18_H_18_S_2_)_2_]	*F*(000) = 688
*M**_r_* = 655.60	*D*_x_ = 1.354 Mg m^−^^3^
Monoclinic, *P*2_1_/*c*	Cu *K*α radiation, λ = 1.54178 Å
*a* = 8.4446 (2) Å	Cell parameters from 9778 reflections
*b* = 14.0911 (2) Å	θ = 6.3–72.3°
*c* = 13.5880 (2) Å	µ = 3.47 mm^−^^1^
β = 96.133 (1)°	*T* = 150 K
*V* = 1607.63 (5) Å^3^	Prism, black
*Z* = 2	0.14 × 0.14 × 0.04 mm

### Bis[1,2-bis(3,5-dimethylphenyl)ethylene-1,2-dithiolato(1-)]nickel(II) Data collection

**Table:**

Bruker D8 QUEST PHOTON 3 diffractometer	3139 independent reflections
Radiation source: INCOATEC IµS micro—-focus source	2827 reflections with *I* > 2σ(*I*)
Mirror monochromator	*R*_int_ = 0.077
Detector resolution: 7.3910 pixels mm^-1^	θ_max_ = 72.4°, θ_min_ = 5.3°
φ and ω scans	*h* = −10→9
Absorption correction: multi-scan (*SADABS*; Krause *et al.*, 2015)	*k* = −17→17
*T*_min_ = 0.77, *T*_max_ = 0.88	*l* = −16→16
33748 measured reflections	

### Bis[1,2-bis(3,5-dimethylphenyl)ethylene-1,2-dithiolato(1-)]nickel(II) Refinement

**Table:**

Refinement on *F*^2^	Primary atom site location: dual
Least-squares matrix: full	Secondary atom site location: difference Fourier map
*R*[*F*^2^ > 2σ(*F*^2^)] = 0.024	Hydrogen site location: inferred from neighbouring sites
*wR*(*F*^2^) = 0.066	H-atom parameters constrained
*S* = 1.08	*w* = 1/[σ^2^(*F*_o_^2^) + (0.0406*P*)^2^ + 0.231*P*] where *P* = (*F*_o_^2^ + 2*F*_c_^2^)/3
3139 reflections	(Δ/σ)_max_ = 0.002
191 parameters	Δρ_max_ = 0.29 e Å^−^^3^
0 restraints	Δρ_min_ = −0.30 e Å^−^^3^

### Bis[1,2-bis(3,5-dimethylphenyl)ethylene-1,2-dithiolato(1-)]nickel(II) Special details

**Table:**

Experimental. The diffraction data were obtained from 18 sets of frames, each of width 0.50 ° in ω or φ, collected with scan parameters determined by the "strategy" routine in *APEX4*. The scan time was 1.00 to 2.00 sec/frame.
Geometry. All esds (except the esd in the dihedral angle between two l.s. planes) are estimated using the full covariance matrix. The cell esds are taken into account individually in the estimation of esds in distances, angles and torsion angles; correlations between esds in cell parameters are only used when they are defined by crystal symmetry. An approximate (isotropic) treatment of cell esds is used for estimating esds involving l.s. planes.
Refinement. Refinement of F^2^ against ALL reflections. The weighted R-factor wR and goodness of fit S are based on F^2^, conventional R-factors R are based on F, with F set to zero for negative F^2^. The threshold expression of F^2^ > 2sigma(F^2^) is used only for calculating R-factors(gt) etc. and is not relevant to the choice of reflections for refinement. R-factors based on F^2^ are statistically about twice as large as those based on F, and R- factors based on ALL data will be even larger.

### Bis[1,2-bis(3,5-dimethylphenyl)ethylene-1,2-dithiolato(1-)]nickel(II) Fractional atomic coordinates and isotropic or equivalent isotropic displacement parameters (Å^2^)

**Table:**

	*x*	*y*	*z*	*U*_iso_*/*U*_eq_	
Ni1	0.000000	1.000000	0.500000	0.01729 (9)	
S1	0.19671 (3)	0.95781 (2)	0.60078 (2)	0.02015 (9)	
S2	−0.00937 (3)	0.86456 (2)	0.43113 (2)	0.01909 (9)	
C1	0.23361 (13)	0.84300 (9)	0.57198 (9)	0.0178 (2)	
C2	0.36518 (14)	0.79450 (8)	0.63520 (8)	0.0185 (2)	
C3	0.52354 (14)	0.81955 (9)	0.62757 (9)	0.0204 (2)	
H3	0.547341	0.869239	0.584243	0.025*	
C4	0.64722 (14)	0.77144 (10)	0.68374 (9)	0.0227 (2)	
C5	0.60798 (14)	0.70147 (9)	0.74937 (9)	0.0220 (2)	
H5	0.691341	0.669344	0.788443	0.026*	
C6	0.45082 (14)	0.67694 (9)	0.75972 (9)	0.0205 (2)	
C7	0.32915 (14)	0.72416 (9)	0.70087 (9)	0.0198 (2)	
H7	0.221118	0.707946	0.705910	0.024*	
C8	0.81906 (16)	0.79350 (13)	0.67185 (11)	0.0347 (3)	
H8A	0.884086	0.784314	0.735298	0.052*	
H8B	0.828065	0.859488	0.650366	0.052*	
H8C	0.856592	0.751012	0.622164	0.052*	
C9	0.41374 (16)	0.60121 (10)	0.83200 (10)	0.0267 (3)	
H9A	0.299904	0.602821	0.840280	0.040*	
H9B	0.475912	0.612633	0.896001	0.040*	
H9C	0.441270	0.538868	0.806746	0.040*	
C10	0.14043 (13)	0.79976 (8)	0.49382 (8)	0.0175 (2)	
C11	0.16772 (14)	0.70341 (9)	0.45561 (9)	0.0194 (2)	
C12	0.32241 (15)	0.67305 (9)	0.44488 (9)	0.0209 (2)	
H12	0.409948	0.712794	0.466938	0.025*	
C13	0.35040 (16)	0.58592 (9)	0.40260 (9)	0.0235 (2)	
C14	0.22139 (17)	0.52790 (9)	0.37169 (9)	0.0274 (3)	
H14	0.239969	0.467764	0.343480	0.033*	
C15	0.06530 (17)	0.55596 (10)	0.38116 (10)	0.0265 (3)	
C16	0.03943 (15)	0.64417 (9)	0.42290 (9)	0.0230 (2)	
H16	−0.066303	0.664398	0.429274	0.028*	
C17	0.51798 (17)	0.55730 (11)	0.38711 (11)	0.0317 (3)	
H17A	0.524013	0.488055	0.381859	0.048*	
H17B	0.591452	0.578953	0.443336	0.048*	
H17C	0.547411	0.586274	0.326076	0.048*	
C18	−0.0720 (2)	0.49193 (11)	0.34582 (13)	0.0379 (3)	
H18A	−0.157674	0.529667	0.310700	0.057*	
H18B	−0.111446	0.460583	0.402777	0.057*	
H18C	−0.036098	0.443897	0.300991	0.057*	

### Bis[1,2-bis(3,5-dimethylphenyl)ethylene-1,2-dithiolato(1-)]nickel(II) Atomic displacement parameters (Å^2^)

**Table:**

	*U*^11^	*U*^22^	*U*^33^	*U*^12^	*U*^13^	*U*^23^
Ni1	0.01736 (15)	0.01340 (15)	0.01985 (15)	0.00354 (9)	−0.00388 (11)	−0.00123 (10)
S1	0.02112 (15)	0.01491 (15)	0.02242 (15)	0.00378 (9)	−0.00688 (11)	−0.00326 (10)
S2	0.01952 (15)	0.01512 (14)	0.02085 (14)	0.00370 (9)	−0.00609 (10)	−0.00226 (10)
C1	0.0185 (5)	0.0148 (5)	0.0197 (5)	0.0022 (4)	0.0003 (4)	0.0000 (4)
C2	0.0192 (5)	0.0171 (5)	0.0180 (5)	0.0042 (4)	−0.0035 (4)	−0.0030 (4)
C3	0.0218 (6)	0.0206 (6)	0.0180 (5)	0.0002 (4)	−0.0020 (4)	−0.0008 (4)
C4	0.0188 (5)	0.0277 (6)	0.0207 (5)	0.0023 (5)	−0.0025 (4)	−0.0037 (5)
C5	0.0198 (6)	0.0252 (6)	0.0198 (5)	0.0083 (4)	−0.0039 (4)	−0.0017 (5)
C6	0.0246 (6)	0.0188 (6)	0.0175 (5)	0.0063 (4)	−0.0001 (4)	−0.0011 (4)
C7	0.0184 (5)	0.0184 (5)	0.0218 (5)	0.0027 (4)	−0.0015 (4)	−0.0013 (4)
C8	0.0205 (6)	0.0495 (9)	0.0334 (7)	−0.0008 (6)	−0.0012 (5)	0.0055 (7)
C9	0.0289 (6)	0.0257 (6)	0.0251 (6)	0.0056 (5)	0.0009 (5)	0.0052 (5)
C10	0.0167 (5)	0.0158 (5)	0.0194 (5)	0.0027 (4)	−0.0008 (4)	0.0013 (4)
C11	0.0248 (6)	0.0155 (6)	0.0168 (5)	0.0036 (4)	−0.0019 (4)	0.0006 (4)
C12	0.0245 (6)	0.0184 (6)	0.0191 (5)	0.0036 (4)	−0.0008 (4)	0.0011 (4)
C13	0.0326 (6)	0.0203 (6)	0.0178 (5)	0.0074 (5)	0.0036 (5)	0.0027 (4)
C14	0.0437 (8)	0.0167 (6)	0.0218 (6)	0.0046 (5)	0.0030 (5)	−0.0028 (5)
C15	0.0358 (7)	0.0192 (6)	0.0237 (6)	−0.0023 (5)	−0.0006 (5)	−0.0029 (5)
C16	0.0252 (6)	0.0188 (6)	0.0243 (6)	0.0006 (4)	−0.0008 (5)	−0.0014 (5)
C17	0.0381 (7)	0.0276 (7)	0.0311 (7)	0.0123 (6)	0.0114 (6)	0.0006 (5)
C18	0.0447 (8)	0.0272 (7)	0.0411 (8)	−0.0108 (6)	0.0004 (7)	−0.0107 (6)

### Bis[1,2-bis(3,5-dimethylphenyl)ethylene-1,2-dithiolato(1-)]nickel(II) Geometric parameters (Å, º)

**Table:**

Ni1—S1^i^	2.1218 (3)	C9—H9A	0.9800
Ni1—S1	2.1218 (3)	C9—H9B	0.9800
Ni1—S2^i^	2.1233 (3)	C9—H9C	0.9800
Ni1—S2	2.1233 (3)	C10—C11	1.4804 (16)
S1—C1	1.7010 (12)	C11—C12	1.3969 (17)
S2—C10	1.7115 (11)	C11—C16	1.4018 (18)
C1—C10	1.3927 (16)	C12—C13	1.3865 (18)
C1—C2	1.4952 (15)	C12—H12	0.9500
C2—C7	1.3889 (17)	C13—C14	1.391 (2)
C2—C3	1.3976 (17)	C13—C17	1.5077 (18)
C3—C4	1.4009 (17)	C14—C15	1.395 (2)
C3—H3	0.9500	C14—H14	0.9500
C4—C5	1.3929 (19)	C15—C16	1.3932 (18)
C4—C8	1.5095 (18)	C15—C18	1.5064 (19)
C5—C6	1.3931 (18)	C16—H16	0.9500
C5—H5	0.9500	C17—H17A	0.9800
C6—C7	1.4009 (17)	C17—H17B	0.9800
C6—C9	1.5057 (17)	C17—H17C	0.9800
C7—H7	0.9500	C18—H18A	0.9800
C8—H8A	0.9800	C18—H18B	0.9800
C8—H8B	0.9800	C18—H18C	0.9800
C8—H8C	0.9800		
			
S1^i^—Ni1—S1	180.0	H9A—C9—H9B	109.5
S1^i^—Ni1—S2^i^	91.279 (10)	C6—C9—H9C	109.5
S1—Ni1—S2^i^	88.723 (10)	H9A—C9—H9C	109.5
S1^i^—Ni1—S2	88.721 (10)	H9B—C9—H9C	109.5
S1—Ni1—S2	91.278 (10)	C1—C10—C11	124.90 (10)
S2^i^—Ni1—S2	180.0	C1—C10—S2	118.26 (9)
C1—S1—Ni1	105.55 (4)	C11—C10—S2	116.69 (8)
C10—S2—Ni1	105.63 (4)	C12—C11—C16	118.96 (11)
C10—C1—C2	124.25 (11)	C12—C11—C10	119.99 (11)
C10—C1—S1	119.28 (9)	C16—C11—C10	120.89 (11)
C2—C1—S1	116.46 (9)	C13—C12—C11	121.14 (12)
C7—C2—C3	120.32 (10)	C13—C12—H12	119.4
C7—C2—C1	119.67 (11)	C11—C12—H12	119.4
C3—C2—C1	120.01 (11)	C12—C13—C14	118.88 (12)
C2—C3—C4	120.06 (12)	C12—C13—C17	119.92 (13)
C2—C3—H3	120.0	C14—C13—C17	121.15 (12)
C4—C3—H3	120.0	C13—C14—C15	121.53 (12)
C5—C4—C3	118.48 (11)	C13—C14—H14	119.2
C5—C4—C8	120.76 (11)	C15—C14—H14	119.2
C3—C4—C8	120.75 (12)	C16—C15—C14	118.76 (12)
C4—C5—C6	122.32 (11)	C16—C15—C18	121.02 (13)
C4—C5—H5	118.8	C14—C15—C18	120.22 (13)
C6—C5—H5	118.8	C15—C16—C11	120.72 (12)
C5—C6—C7	118.23 (12)	C15—C16—H16	119.6
C5—C6—C9	120.56 (11)	C11—C16—H16	119.6
C7—C6—C9	121.20 (11)	C13—C17—H17A	109.5
C2—C7—C6	120.53 (11)	C13—C17—H17B	109.5
C2—C7—H7	119.7	H17A—C17—H17B	109.5
C6—C7—H7	119.7	C13—C17—H17C	109.5
C4—C8—H8A	109.5	H17A—C17—H17C	109.5
C4—C8—H8B	109.5	H17B—C17—H17C	109.5
H8A—C8—H8B	109.5	C15—C18—H18A	109.5
C4—C8—H8C	109.5	C15—C18—H18B	109.5
H8A—C8—H8C	109.5	H18A—C18—H18B	109.5
H8B—C8—H8C	109.5	C15—C18—H18C	109.5
C6—C9—H9A	109.5	H18A—C18—H18C	109.5
C6—C9—H9B	109.5	H18B—C18—H18C	109.5
			
Ni1—S1—C1—C10	1.02 (11)	C2—C1—C10—S2	178.12 (9)
Ni1—S1—C1—C2	−177.97 (8)	S1—C1—C10—S2	−0.78 (14)
C10—C1—C2—C7	−70.01 (16)	Ni1—S2—C10—C1	0.13 (11)
S1—C1—C2—C7	108.92 (11)	Ni1—S2—C10—C11	−175.73 (8)
C10—C1—C2—C3	109.54 (14)	C1—C10—C11—C12	−41.64 (18)
S1—C1—C2—C3	−71.52 (13)	S2—C10—C11—C12	133.91 (10)
C7—C2—C3—C4	2.22 (18)	C1—C10—C11—C16	143.13 (12)
C1—C2—C3—C4	−177.33 (11)	S2—C10—C11—C16	−41.32 (15)
C2—C3—C4—C5	−2.46 (18)	C16—C11—C12—C13	0.06 (18)
C2—C3—C4—C8	176.27 (12)	C10—C11—C12—C13	−175.26 (11)
C3—C4—C5—C6	1.02 (19)	C11—C12—C13—C14	−0.76 (18)
C8—C4—C5—C6	−177.71 (13)	C11—C12—C13—C17	176.79 (11)
C4—C5—C6—C7	0.68 (19)	C12—C13—C14—C15	0.83 (19)
C4—C5—C6—C9	−179.67 (12)	C17—C13—C14—C15	−176.69 (13)
C3—C2—C7—C6	−0.47 (18)	C13—C14—C15—C16	−0.2 (2)
C1—C2—C7—C6	179.08 (11)	C13—C14—C15—C18	179.27 (13)
C5—C6—C7—C2	−0.96 (18)	C14—C15—C16—C11	−0.5 (2)
C9—C6—C7—C2	179.39 (11)	C18—C15—C16—C11	−179.98 (13)
C2—C1—C10—C11	−6.39 (19)	C12—C11—C16—C15	0.60 (18)
S1—C1—C10—C11	174.70 (9)	C10—C11—C16—C15	175.88 (12)

Symmetry code: (i) −*x*, −*y*+2, −*z*+1.
